# Observation of dressed states of distant atoms with delocalized photons in coupled-cavities quantum electrodynamics

**DOI:** 10.1038/s41467-019-08975-8

**Published:** 2019-03-11

**Authors:** Shinya Kato, Nikolett Német, Kohei Senga, Shota Mizukami, Xinhe Huang, Scott Parkins, Takao Aoki

**Affiliations:** 1 Department of Applied Physics, Waseda University, 3-4-1 Okubo, Shinjuku, Tokyo 169-8555 Japan; 2 JST, PRESTO, 4-1-8 Honcho, Kawaguchi, Saitama 332-0012 Japan; 3 The Dodd-Walls Centre for Photonic and Quantum Technologies, Dunedin, New Zealand; 4 Department of Physics, University of Auckland, Auckland, 1010 New Zealand

## Abstract

In a cavity quantum electrodynamics (QED) system, where atoms coherently interact with photons in a cavity, the eigenstates of the system are the superposition states of atoms and cavity photons, the so-called dressed states of atoms. When two cavities are connected by an optical fiber with negligible loss, the coherent coupling between the cavities gives rise to photonic normal modes. One of these normal modes is the fiber-dark mode, in which photons are delocalized in the two distant cavities. Here we demonstrate the setting of coupled-cavities QED, where two nanofiber cavity-QED systems are coherently connected by a meter-long low-loss channel in an all-fiber fashion. Specifically, we observe dressed states of distant atoms with delocalized photons of the fiber-dark normal mode. Our system will provide a platform for the study of delocalized atomic and photonic states, photonic many-body physics, and distributed quantum computation.

## Abstract (web summary)

The coherent dynamics of coupled atoms that interact with a common mode is different from that of independent atoms. Here the authors demonstrate the delocalization of excited states of atoms and photons using two ensembles of cold Cs atoms in cavities connected with meter long optical fiber.

## Introduction

When atoms are coherently coupled to each other via their interaction with a common mode of the electromagnetic field, they form collective states, the dynamics of which can be drastically different from that of independent atoms. For the case of atoms in free space, collective effects become observable, as a change in radiative decay rates, when inter-atomic separations are smaller than a few wavelengths^1–4^. Recently, such super-radiance and sub-radiance phenomena have been observed for atoms interacting with a common guided mode of a photonic crystal waveguide^5^ and an optical nanofiber^6^, where atoms are separated by a macroscopic distance up to several hundred microns, much larger than the wavelength. On the other hand, in the setting of cavity quantum electrodynamics (QED), where coherent atom−atom coupling is mediated by the confined mode of the cavity, the collective effects can be observed as coherent, reversible dynamics of the system, which is evident as a structural change in the energy spectra, in contrast to changes in atomic dissipation rates as for the above cases. For example, the vacuum Rabi splitting for the dressed states of an atom in the Jaynes−Cummings model^7–10^ is enhanced by a factor of $\sqrt N$ for the collective dressed states in the *N*-atom Tavis−Cummings model^11–14^. These coherent collective effects may be extended to a configuration of coupled, but distant, cavity QED systems. In particular, when two cavities are connected via a channel whose loss is negligible compared to the coupling between each cavity and the channel, photons can deterministically propagate back and forth between the cavities many times before being lost. Such coherent coupling between two cavities gives rise to normal modes, or superpositions in certain combinations of the two cavities and the connecting fiber, each of which extends to the whole system nonlocally. Notably, one of these normal modes is a superposition of the two cavity modes but has no contribution from the connecting fiber (fiber-dark mode)^15^.

Here, we demonstrate an all-fiber, coupled-cavities QED system in which either a single ensemble or two distantly separated ensembles of several tens of atoms coherently interact with the delocalized normal modes of coherently coupled, distant cavities, and we observe collective dressed states of atoms with the fiber-dark mode. This is the demonstration of coherent, reversible coupling between distant atoms, with a separation of the order of a meter, which is made possible by the all-fiber nature of the connection between the two cavities, with a loss as low as 2%. Our achievement is an important step towards the physical implementation of cavity QED-based distributed quantum computation^15–18^ and a quantum network^19,20^, where a large number of cavity QED systems are coherently connected by low-loss fiber channels. In such systems, quantum entanglement over the whole network can be created deterministically^21,22^, instead of probabilistically^23^. Our achievement also paves the way for the study of many-body physics with atoms and photons in a coupled-cavities QED system, such as quantum phase transitions of light^24–31^.

## Results

### Theory

**Fig. 1:**
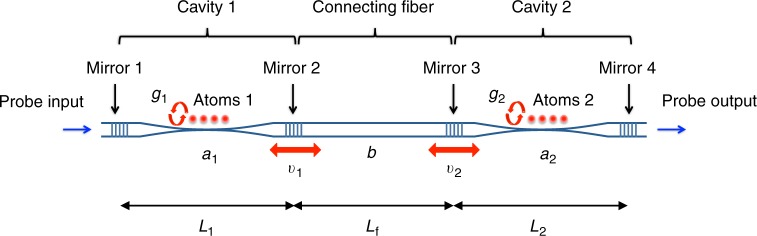
Schematic of the coupled-cavity QED system. Cavity 1 of length *L*_1_ and cavity 2 of length *L*_2_ are coupled to a connecting fiber of length *L*_f_ with coupling rates of *v*_1_ and *v*_2_, respectively. Atomic ensembles are coupled to Cavities 1 and 2 with atom-cavity coupling rates of *g*_1_ and *g*_2_, respectively. Probe beam is input from the left mirror of cavity 1 (Mirror 1), and its output from the right mirror of cavity 2 (Mirror 4) is measured

As illustrated in Fig. 1, our system consists of two nanofiber cavity QED systems^32^ connected in an all-fiber fashion. In each cavity, an ensemble of several tens of atoms interacts with the cavity field through the evanescent field of a nanofiber, both ends of which are connected to standard optical fibers through tapered regions and sandwiched by a pair of fiber-Bragg-grating mirrors. A probe laser at the frequency *ω*_p_ is input from mirror 1 (left mirror of cavity 1), and the output from mirror 4 (right mirror of cavity 2) is measured.

For simplicity, we first consider the system with one atom for each cavity, which is modeled by the following Hamiltonian (ℏ = 1) in a frame rotating at *ω*_p_:

1 $$\begin{array}{*{20}{l}} H \hfill & = \hfill & {{\mathrm{\Delta }}_{\mathrm{c}}\left( {a_1^\dagger a_1 + a_2^\dagger a_2 + b^\dagger b} \right) + \mathop {\sum}\limits_{i = 1,2} v_i\left( {a_i^\dagger b + b^\dagger a_i} \right)} \hfill \\ {} \hfill & {} \hfill & { + \,{\mathrm{\Delta }}_{\mathrm{a}}\left( {\sigma _1^ + \sigma _1^ - + \sigma _2^ + \sigma _2^ - } \right) + \mathop {\sum}\limits_{i = 1,2} g_i\left( {a_i^\dagger \sigma _i^ - + \sigma _i^ + a_i} \right),} \hfill \end{array}$$

where we assume, for simplicity, that the cavity and connecting-fiber modes (*a*_1_, *a*_2_, *b*) have the same frequency *ω*_c_ so that Δ_c_ = *ω*_c_ − *ω*_p_ (see Supplementary Note 1). The atom-probe detuning is given by Δ_a_ = *ω*_a_ − *ω*_p_, where *ω*_a_ is the atomic transition frequency. The coupling rates of cavities 1 and 2 with the connecting fiber are given by

2 $$v_{1,2} = \frac{c}{2}\sqrt {\frac{{T_{2,3}}}{{L_{\mathrm{f}}L_{1,2}}}} ,$$

where *c* is the speed of light in the fiber and *T*_*i*_, *L*_*i*_, and *L*_f_ are the transmittance of the mirror *i*, length of the cavity *i*, and length of the connecting fiber, respectively. The atoms are coupled to their respective cavity modes with strengths *g*_1_ and *g*_2_.

Considering just the cavities and connecting fiber, we can move to a normal-mode picture, with normal-mode operators given by

3 $$d = \frac{1}{{\sqrt 2 \tilde v}}(v_2a_1 + v_1a_2),$$

4 $$c_ \pm = \frac{1}{{2\tilde v}}(v_1a_1 + v_2a_2) \pm \frac{1}{{\sqrt 2 }}b,$$

where

5 $$\tilde v = \sqrt {\frac{{v_1^2 + v_2^2}}{2}} .$$

Note that the normal mode *d* has no contribution from the connecting-fiber mode *b*. Rather, it has only contributions from the two cavity modes *a*_1_ and *a*_2_, and it has the frequency *ω*_c_. On the other hand, the normal modes *c*_±_ have contributions from the two cavities and the connecting fiber, and they are shifted in frequency by $\pm \sqrt 2 \tilde v$ from *ω*_c_. If $\sqrt 2 \tilde v$ is sufficiently large, the atomic transition frequency is close to the bare cavity resonance, $\omega _{\mathrm{a}} \simeq \omega _{\mathrm{c}}$, and the frequency of the probe laser is scanned only in this vicinity, then it is possible to focus on the system dynamics involving only the mode *d*. That is, we can focus on the reduced Hamiltonian

6 $$H_d = {\mathrm{\Delta }}_{\mathrm{c}}d^\dagger d + \mathop {\sum}\limits_{i = 1,2} \left[ {{\mathrm{\Delta }}_{\mathrm{a}}\sigma _i^ + \sigma _i^ - + g_{di}\left( {d^\dagger \sigma _i^ - + \sigma _i^ + d} \right)} \right],$$

where

7 $$g_{d1,d2} = \frac{{v_{2,1}}}{{\sqrt 2 \tilde v}}g_{1,2}.$$

This Hamiltonian is identical to the Hamiltonian for a standard single-cavity QED system with a cavity mode *d* and two atoms (Tavis−Cummings Hamiltonian^11^) having single-atom coupling strengths of *g*_*d*1_ and *g*_*d*2_. The eigenstates of this system are the collective dressed states of atoms in distant cavities and of photons in the delocalized normal mode *d*. In other words, each atom interacts with both cavities simultaneously and collectively, but not with the connecting fiber. Indeed, the atom-field coupling strengths, *g*_*d*1_ and *g*_*d*2_, do not depend on the length of the connecting fiber.

For a general case of the system with many atoms in each cavity, the linear optical response in the weak-driving limit is identical to the single-atom model discussed above, with replacements of single-atom coupling strengths *g*_*i*_ with the collective coupling strengths $g_{i,{\mathrm{eff}}} = g_{i,(0)}\sqrt {N_{i,{\mathrm{eff}}}}$, where *g*_*i*,(0)_ is the single-atom coupling strength for an atom located at a potential minimum of the atomic trap in cavity *i* and *N*_*i*,eff_ is the effective number of atoms in cavity *i* (see Supplementary Note 1).

### Experiments

In order to investigate the interaction between atoms and the normal modes of the coupled-cavities system, we measure transmission spectra with different atom-loading conditions and various lengths of the connecting fiber.

Each cavity QED system is similar to the previous setup described in ref. ^32^. The transmittances of the mirrors are (*T*_1_, *T*_2_, *T*_3_, *T*_4_) = (0.13, 0.39, 0.33, 0.06). The single-pass losses inside the cavities and that for the connecting fiber are all 0.02, which are dominated by the splicing losses (two splices for each cavity and the connecting fiber). The cavity lengths are (*L*_1_, *L*_2_) = (0.92, 1.38) m. Atoms are trapped in a state-insensitive, two-color, evanescent-field optical trap^32–36^.

**Fig. 2:**
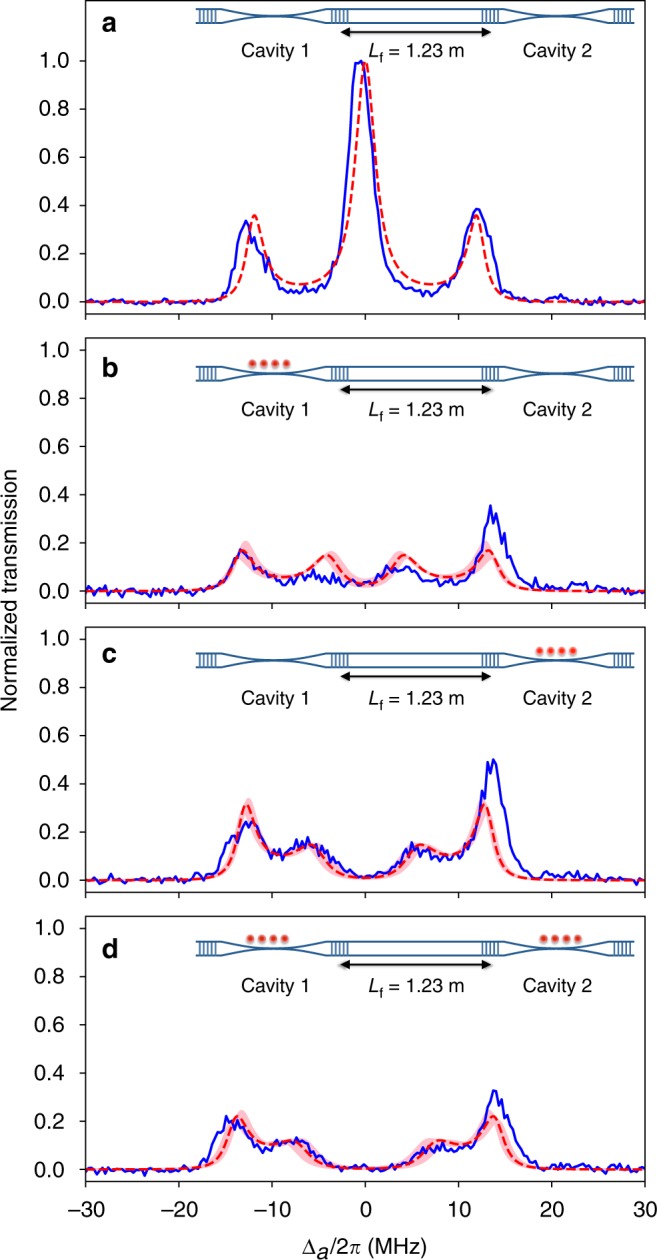
Transmission spectra with different atom-loading conditions. **a** No atoms are loaded. **b** Atoms are loaded in cavity 1 only. **c** Atoms are loaded in cavity 2 only. **d** Atoms are loaded in both cavities. Blue solid lines represent experimental data, while red dashed lines and pink shaded bands are theoretical curves for (*g*_1,eff_, *g*_2,eff_) = 2*π* × (7.2, 7.3) MHz and (*g*_1,eff_, *g*_2,eff_) = 2*π* × (7.2 ± 1.0, 7.3 ± 1.0) MHz, respectively. When no atoms are loaded (**a**), formation of normal modes for three coupled empty cavities results in the clear triplet spectrum. The central peak corresponds to the fiber-dark mode *d*, while the two side peaks correspond to the other two normal modes *c*_±_. When atoms are loaded in one of the two (**b**, **c**), or both cavities (**d**), coupling between atoms and the fiber-dark mode results in the splitting of the central peak observed in (**a**). In particular, the splitting observed in (**d**) is the signature of the dressed states of distant atoms with delocalized photons in the fiber-dark mode

Firstly, we fix the length of the connecting fiber as *L*_f_ = 1.23 m and measure the transmission spectra of the coupled-cavities QED system with different atom-loading conditions, in which the atoms are not loaded, loaded in either cavity, or loaded in both cavities. In Fig. 2a, the blue solid line shows the measured transmission spectrum normalized to the maximum transmission for the case without atoms. Three peaks for the normal modes *d* and *c*_±_ are clearly observed. The red dashed line shows the corresponding theoretical curve (see Supplementary Note 1) with no free fitting parameter, which agrees well with the experimental data. In particular, the splittings of the side peaks from the center peak match with the theoretical value of the frequency difference of the modes *c*_±_ from the fiber-dark mode *d* given by $\sqrt 2 \tilde v = 2\pi \times 12.1\,{\mathrm{MHz}}$. The slight broadening in linewidths and the asymmetry of the spectral shape in the experiments are, we believe, due to the instability of the detunings of the cavity and fiber modes (*a*_1_, *a*_2_, and *b*) from the atomic frequency *ω*_a_ during the measurement. Figure 2b, c shows the spectra for the cases of atoms loaded only in cavities 1 and 2, respectively. It can be clearly seen that the interaction of atoms with the fiber-dark mode *d* causes a splitting of the center peak in Fig. 2a, which is the signature of the dressed states of atoms with delocalized photons of the fiber-dark mode. On the other hand, the (off-resonant) interaction of atoms with the modes *c*_±_ causes the frequency shifts of the side peaks. It can be seen that the spectra agree reasonably well with the theoretical curves of a linearized model with the atom-cavity coupling strengths (*g*_1,eff_, *g*_2,eff_) = 2*π* × (7.2 ± 1.0, 7.3 ± 1.0) MHz ((*g*_*d*1_, *g*_*d*2_) = 2*π* × (4.3 ± 0.6, 5.8 ± 0.8) MHz) as the only free parameters. Figure 2d shows the spectrum for the case of atoms loaded in both cavities. The measured spectra clearly also agree quite well with the theoretical curves with no additional free parameters. Note that the splitting of the center peak is larger than that observed in Fig. 2b, c and agrees with the theoretical value of $\sqrt {g_{d1}^2 + g_{d2}^2} = 2\pi \times 7.3\,{\mathrm{MHz}}$, which is the signature of the collective dressed states of distant atoms with delocalized photons of the fiber-dark mode.

**Fig. 3:**
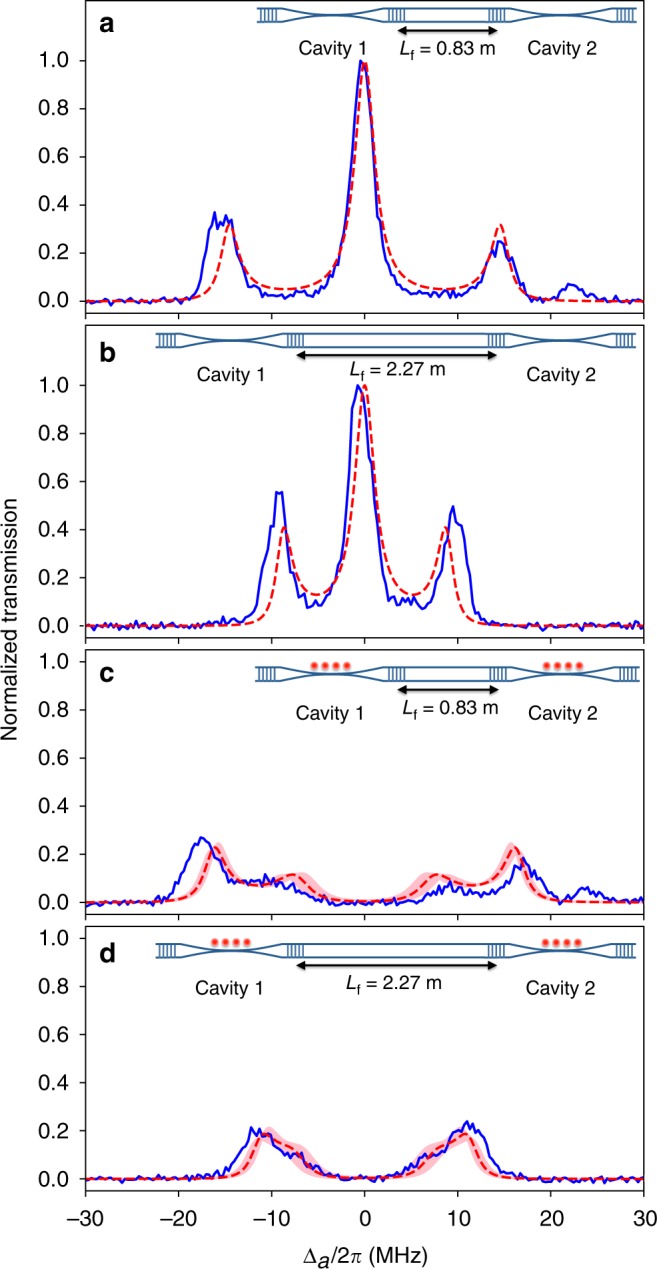
Transmission spectra with different connecting-fiber lengths. Blue solid lines represent experimental data, while red dashed lines and pink shaded bands are theoretical curves for (*g*_1,eff_, *g*_2,eff_) = 2*π* × (7.2, 7.3) MHz and (*g*_1,eff_, *g*_2,eff_) = 2*π* × (7.2 ± 1.0, 7.3 ± 1.0) MHz, respectively. The small peaks at Δ_a_ ≈ 2*π* × 25 MHz in (**a**, **c**) correspond to a mode with different polarization, which appears because of imperfect polarization compensation in the connecting fiber (see Supplementary Note 2). The dependence of the cavity-fiber coupling rates *v*_1_ and *v*_2_ on the connecting-fiber length is clearly observed as the change of the splitting in the triplet structure in (**a**, **b**). In contrast, the splitting of the central peak in (**c**, **d**) remains unchanged. This is because the coupling rate between atoms and the delocalized photons in the fiber-dark mode does not depend on the connecting-fiber length

Secondly, we measure transmission spectra of the coupled-cavities QED system with different lengths of the connecting fiber. Figure 3a, b shows the spectra without atoms for the cases of *L*_f_ = 0.83 m and 2.27 m, respectively. The measured spectra (blue solid lines) reasonably agree with the theoretical curves (red dashed lines). A change of splittings between the center and side peaks from that in Fig. 2a (*L*_f_ = 1.23 m) is clearly observed. Specifically, the observed splittings roughly match the theoretical values of $\sqrt 2 \tilde v = 2\pi \times 14.7\,{\mathrm{MHz}}$ and 2*π* × 8.9 MHz for *L*_f_ = 0.83 m and 2.27 m, respectively. Figure 3c, d shows the spectra with atoms for the cases of *L*_f_ = 0.83 m and 2.27 m, respectively. Again, the measured spectra (blue solid lines) reasonably agree with the theoretical curves (red dashed lines). Note that the splittings of the center peak associated with the coupling of atoms to the fiber-dark mode do not change for different lengths of the connecting fiber, in agreement with the above theory.

**Fig. 4:**
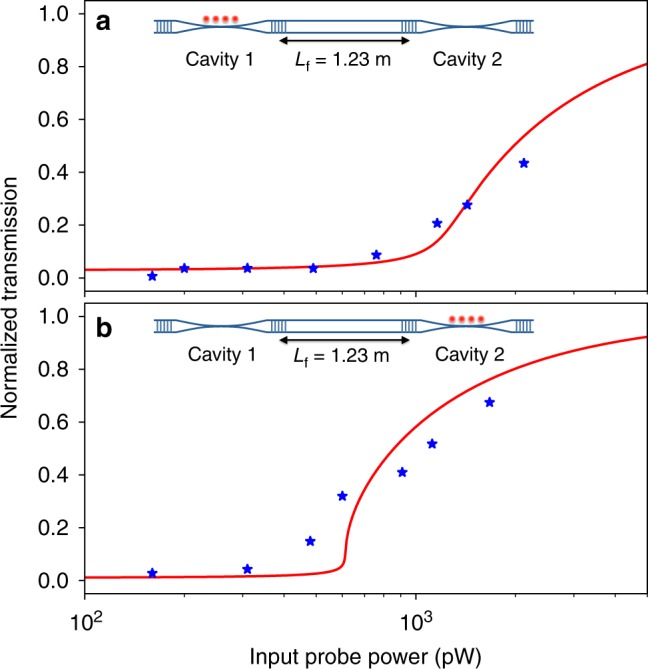
Saturation behavior of the on-resonance transmission. Normalized transmission is plotted as a function of input probe power for atoms loaded in **a** cavity 1 and **b** cavity 2. Red curves are semiclassical state equations with (*g*_1,(0)_, *g*_2,(0)_) = 2*π* × (0.75, 1.2) MHz and (*N*_1,eff_, *N*_2,eff_) = (92, 37). The corresponding saturation photon numbers are (*n*_1,sat_, *n*_2,sat_) = (6.9, 2.7), and thus, our system shows strong nonlinearity at the few-photon level

Lastly, we investigate saturation behaviors of the coupled-cavities QED system to confirm that the above measurements are conducted in the weak-driving regime. Specifically, we load atoms in either of the cavities and measure transmission as a function of the input probe power. The length of the connecting fiber is fixed at *L*_f_ = 1.23 m. Figure 4a, b shows the normalized transmission at zero detuning Δ_a_ = 0 for atoms loaded in cavities 1 and 2, respectively. It can be clearly seen that the system is in the weak-driving regime at the input power of 210–310 pW for the above measurement and that the transmission starts to saturate as the input power exceeds ~10^3^ pW.

We further compare the measured saturation behaviors with semiclassical state equations for the many-atom coupled-cavities QED system (see Supplementary Note 1). The red solid lines in Fig. 4a, b are the theoretical curves with (*g*_1,(0)_, *g*_2,(0)_) = 2*π* × (0.75, 1.2) MHz, from which we obtain (*N*_1,eff_, *N*_2,eff_) ≡ ((*g*_1,eff_/*g*_1,(0)_)^2^, (*g*_2,eff_/*g*_2,(0)_)^2^) = (92, 37). It can also be seen that these curves reasonably agree with the experimental results. Saturation behavior of a cavity QED system can be characterized with the so-called saturation photon number, which is a measure of the system nonlinearity. Note that the corresponding saturation photon numbers are (*n*_1,sat_, *n*_2,sat_) = (6.9, 2.7) (see Supplementary Note 1), and thus, our system shows strong nonlinearity at the few-photon level.

## Discussion

In summary, we have demonstrated the setting of a coupled-cavities QED system in which two nanofiber cavity QED systems are coherently connected via a meter-long, low-loss fiber channel. Specifically, we have observed the collective dressed states of distant atoms with delocalized photons of a fiber-dark normal mode. We note that the delocalization of photons for the fiber-dark mode would be explicitly demonstrated by the inability to drive or detect this mode through the connecting fiber, which could be facilitated by inserting a fiber beam splitter in the connecting fiber. Also, while in the present study the coherent coupling between distant atoms has been observed in the steady-state spectra, it will be possible, and very interesting, to also investigate transient dynamics of this system, where deterministic, reversible exchange of excitation between distant atoms would be observable.

It is straightforward to increase the number of coupled-cavities in our system, and by driving the atoms from the side of the cavities with classical fields, it will be possible to realize a system of strongly interacting polaritons^25^. Since the cavities are coupled via optical fibers, it is also possible to design a system with arbitrary geometry of connections. Note that ensembles of atoms $\gtrsim 10^3$ are routinely loaded into the optical traps around nanofibers^34,36,37^. By reducing the number of atoms in each cavity to one^32^, on the other hand, it will be possible to construct a fiber network of single-atom-cavity QED systems^19,20^. With such a system, deterministic creation of quantum entanglement over the whole network^21,22^, instead of probabilistic creation^23^, will be possible. Furthermore, provided that the normal-mode description is valid, i.e., if the coherent coupling rate between each cavity and the fiber is larger than the loss rates, the interaction of atoms with the resulting fiber-dark mode can be utilized in quantum gates for distributed quantum computation^15^. In order to extend our work to the construction of a fiber network of coherently coupled single-atom-cavity QED systems, we are currently making technical improvements to the setup, for example, further reduction of the internal losses in the cavities, active stabilization of the cavities, and extending the lifetime of the atomic traps^38^.

## Methods

### Experimental methods

Each experimental sequence starts with cooling and collecting Cs atoms in standard magneto-optical traps (MOTs). We use the D_2_-line F = 4 → F′ = 5 transition for cooling and the F = 3 → F′ = 4 transition for repumping in the MOT. The numbers of atoms in the MOTs are 7 × 10^6^ and 3.3 × 10^7^ for cavities 1 and 2, respectively. The positions of the MOTs are intentionally shifted from the cavities, and atoms are not coupled to the cavities at this stage. After the numbers of atoms in the MOTs are saturated, we scan the lengths of the cavities and the connecting fiber with different frequencies and monitor the transmission of a laser with the frequency *ω*_a_ (atomic F = 4 → F′ = 5 transition). The transmission varies with the lengths of the cavities and the connecting fiber, i.e., the detunings of the modes *a*_1_, *a*_2_, and *b* from *ω*_a_, and takes the maximum value when all the three modes are resonant to *ω*_a_. When the transmission reaches a certain threshold value, we assume that this resonance condition is satisfied and proceed to the next step, in which we switch off the monitor laser and change the detuning and intensity of the cooling laser for 32 ms to further cool the atoms down to 20 μK. Subsequently, we move the MOTs to overlap with the cavities to load atoms in the optical traps in the evanescent fields of the nanofibers.

Atoms are trapped in a state-insensitive, two-color evanescent-field optical trap^32–36^. We use counterpropagating red-detuned beams (*λ*_red_ = 937 nm), which form a one-dimensional optical lattice, and a blue-detuned beam (*λ*_blue_ = 688 nm). The trap depth of the lattice well is about 260 μK.

After loading atoms in the optical traps, we measure the transmission spectrum of the system by sending a probe pulse with the atom-probe detuning Δ_a_ scanned over ±30 MHz within 4 ms and by detecting the transmitted probe beam from the system by an avalanche photodiode after removing unwanted stray light with filters. The power of the input probe is 210–310 pW. Next, we optically pump the atoms into the F = 3 state, which is sufficiently off-resonant from the cavity modes, by irradiating a pumping laser resonant to the atomic F = 4 → F′ = 3 transition from the sides of the cavities for 1 ms, and we send a frequency-scanned probe pulse again to measure the transmission spectrum of the coupled empty cavities, i.e., the system without atoms. We then switch on the cooling and repumping lasers for 4 ms to cool and load the atoms into the optical trap again. We repeat the above procedure for spectroscopy and re-cooling five times per MOT loading sequence, and we use the average of the second, third, and fourth data for each sequence (see Supplementary Note 2 for details).

## Supplementary information

Supplementary Information

Peer Review

## Data Availability

The data that support the findings of this study are available from the corresponding author on reasonable request.
