# Supplementary material for: Observation of dressed states of distant atoms with delocalized photons in coupled-cavities quantum electrodynamics
Source: Nat Commun. 2019 Mar 11;10:1160. doi: 10.1038/s41467-019-08975-8 (PMC6411748; doi:10.1038/s41467-019-08975-8)
Supplement: Supplementary file 1 — Supplementary Information [file 41467_2019_8975_MOESM1_ESM.pdf]

Supplementary Information – Observation of dressed states of distant atoms with delocalized photons in coupled-cavities quantum electrodynamics

Shinya Kato,[1,2] Nikolett Német,[3] Kohei Senga,[1] Shota Mizukami,[1] Xinhe Huang,[1] Scott Parkins,[3] and Takao Aoki[1,*]

[1] *Department of Applied Physics, Waseda University, 3-4-1 Okubo, Shinjuku, Tokyo 169-8555, Japan*
[2] *JST, PRESTO, 4-1-8 Honcho, Kawaguchi, Saitama, 332-0012, Japan*
[3] *Dodd-Walls Centre for Photonic & Quantum Technologies, Department of Physics, University of Auckland, Private Bag 92019, Auckland 1142, New Zealand*

Supporting information for our manuscript "Coupled-cavities quantum electrodynamics: observation of dressed states of distant atoms with delocalized photons" is provided.

Supplementary Note 1: Theory

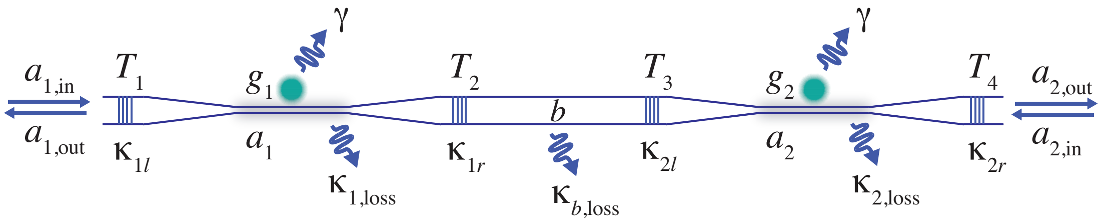

Supplementary Fig. 1. Schematic of the coupled-cavities system (not to scale). The transmittance of mirror i is T_i. Other parameters are defined in the text.

1. *Coupled-cavities system*

As a simple model of our system (Supplementary Fig. 1), we can use single modes for the fields in the cavities and in the connecting fiber, as well as single, two-level atoms in each cavity, and a master equation for the density operator ρ of the composite system (atoms plus fields) that takes the form (in a frame rotating at the probe laser frequency ω_p, and setting $\hbar = 1$)

$$\dot{\rho} = -i[H,\rho] + \kappa_1 \mathcal{D}[a_1]\rho + \kappa_2 \mathcal{D}[a_2]\rho + \kappa_{b,\mathrm{loss}}\mathcal{D}[b]\rho + \frac{\gamma_\parallel}{2}\left(\mathcal{D}[\sigma_1^-]\rho + \mathcal{D}[\sigma_2^-]\rho\right)$$
$$+ \gamma_\mathrm{las}\left(\mathcal{D}[a_1^\dagger a_1]\rho + \mathcal{D}[a_2^\dagger a_2]\rho + \mathcal{D}[b^\dagger b]\rho + \mathcal{D}[\sigma_{z,1}]\rho + \mathcal{D}[\sigma_{z,2}]\rho\right). \quad (1)$$

where $\mathcal{D}[O]\rho = 2O\rho O^\dagger - O^\dagger O\rho - \rho O^\dagger O$. The Hamiltonian is

$$H = \Delta_\mathrm{c}\left(a_1^\dagger a_1 + a_2^\dagger a_2 + b^\dagger b\right) + \left(v_1^* a_1^\dagger b + v_1 b^\dagger a_1\right) + \left(v_2^* a_2^\dagger b + v_2 b^\dagger a_2\right) + \left(\mathcal{E}_\mathrm{p}^* a_1 + \mathcal{E}_\mathrm{p} a_1^\dagger\right)$$
$$+ \Delta_\mathrm{a}\left(\sigma_1^+\sigma_1^- + \sigma_2^+\sigma_2^-\right) + \left(g_1 a_1^\dagger \sigma_1^- + g_1^* \sigma_1^+ a_1\right) + \left(g_2 a_2^\dagger \sigma_2^- + g_2^* \sigma_2^+ a_2\right), \quad (2)$$

where we assume, for simplicity, that the cavity and connecting-fiber modes (a_1, a_2, b) have the same frequency ω_c, so that $\Delta_\mathrm{c} = \omega_\mathrm{c} - \omega_\mathrm{p}$. The atom-probe detuning is $\Delta_\mathrm{a} = \omega_\mathrm{a} - \omega_\mathrm{p}$, where ω_a is the atomic transition frequency, and $\mathcal{E}_\mathrm{p}$ is the probe driving strength. The atoms couple with strengths $g_{1,2}$ to their respective cavity modes, while the coupling rates between the cavity modes (of lengths $L_{1,2}$) and the fiber mode (of length L_f) are given by

$$v_1 = \sqrt{\frac{\kappa_{1r}}{\pi}\omega_{\mathrm{FSR,f}}} \equiv \frac{c}{2}\sqrt{\frac{T_2}{L_1 L_\mathrm{f}}} \quad \text{and} \quad v_2 = \sqrt{\frac{\kappa_{2l}}{\pi}\omega_{\mathrm{FSR,f}}} \equiv \frac{c}{2}\sqrt{\frac{T_3}{L_2 L_\mathrm{f}}}. \quad (3)$$

* takao@waseda.jp

Here, $\omega_{\mathrm{FSR,f}} = \pi c/L_{\mathrm{f}}$ is the free spectral range of the coupling fiber mode, where c is the speed of light in the fiber, and $\kappa_{1r} = cT_2/(4L_1)$ and $\kappa_{2l} = cT_3/(4L_2)$ correspond to the decay rates of the respective cavity fields through mirrors 2 and 3 in the case that the outputs from these mirrors couple to a continuum of modes (for example, in the limit that $L_{\mathrm{f}} \to \infty$).

The remaining terms in the master equation describe losses and dephasing effects in the system. The fiber mode b has an intrinsic loss rate $\kappa_{b,\mathrm{loss}}$, while the field decay rates of cavities 1 and 2 are given by

$$\kappa_1 = \kappa_{1l} + \kappa_{1,\mathrm{loss}} \quad \text{and} \quad \kappa_2 = \kappa_{2r} + \kappa_{2,\mathrm{loss}}, \tag{4}$$

where $\kappa_{1l} = cT_1/(4L_1)$ and $\kappa_{2r} = cT_4/(4L_2)$. The intrinsic loss rates are determined from the (intensity) transmission coefficients of the fiber segments that support the various modes as

$$\kappa_{1,\mathrm{loss}} = -\frac{1}{2}\frac{c}{L_1}\ln(1-\alpha_1), \quad \kappa_{b,\mathrm{loss}} = -\frac{1}{2}\frac{c}{L_{\mathrm{f}}}\ln(1-\alpha_{\mathrm{f}}), \quad \kappa_{2,\mathrm{loss}} = -\frac{1}{2}\frac{c}{L_2}\ln(1-\alpha_2), \tag{5}$$

where α_1, α_{f}, α_2 are single-pass losses for the segments in cavity 1, the connecting fiber, and cavity 2, respectively. The term proportional to γ_{las} – the laser linewidth (HWHM) – is included so as to incorporate the effect of laser frequency fluctuations, which appears as phase damping of the field and atomic amplitudes. The atoms decay into free space with rate $\gamma_{\|}$.

Parameter	$2\pi\cdot$MHz
κ_{1l}	1.16
$\kappa_{1,\mathrm{loss}}$	0.36
κ_{1r}	3.48
κ_{2l}	1.97
$\kappa_{2,\mathrm{loss}}$	0.24
κ_{2r}	0.357
$\kappa_{b,\mathrm{loss}}$ ($L_{\mathrm{f}} = 0.83\,\mathrm{m}$)	0.40
$\kappa_{b,\mathrm{loss}}$ ($L_{\mathrm{f}} = 1.23\,\mathrm{m}$)	0.27
$\kappa_{b,\mathrm{loss}}$ ($L_{\mathrm{f}} = 2.27\,\mathrm{m}$)	0.15
v_1 ($L_{\mathrm{f}} = 0.83\,\mathrm{m}$)	11.7
v_1 ($L_{\mathrm{f}} = 1.23\,\mathrm{m}$)	9.65
v_1 ($L_{\mathrm{f}} = 2.27\,\mathrm{m}$)	7.10
v_2 ($L_{\mathrm{f}} = 0.83\,\mathrm{m}$)	8.82
v_2 ($L_{\mathrm{f}} = 1.23\,\mathrm{m}$)	7.25
v_2 ($L_{\mathrm{f}} = 2.27\,\mathrm{m}$)	5.33
$\gamma_{\|}$	5.2
γ_{las}	0.365

Supplementary Table I. List of parameter values for modeling of the experiment. κ_{1l}, κ_{1r}, κ_{2l}, κ_{2r} correspond to the decay rates of the respective cavity fields through mirrors 1, 2, 3, and 4 (in the case that the outputs from these mirrors couple to a continuum of modes). $\kappa_{1,\mathrm{loss}}$, $\kappa_{b,\mathrm{loss}}$, $\kappa_{2,\mathrm{loss}}$ are the intrinsic loss rates for cavity 1, connecting fiber, and cavity 2. v_1 and v_2 are the coupling rates between the cavity modes and the fiber mode. $\gamma_{\|}$ and γ_{las} are the atomic decay rate into free space and the laser linewidth (HWHM), respectively.

2. Weak probe driving: linearized equations of motion

If we assume weak driving and, hence, weak excitation of the atoms, then we may derive the following linear equations of motion for the field and atomic amplitudes:

$$\dot{\langle a_1 \rangle} = -(\kappa_1' + i\Delta_\mathrm{c}) \langle a_1 \rangle - iv_1 \langle b \rangle - ig_1 \langle \sigma_1^- \rangle - i\mathcal{E}_1, \tag{6}$$

$$\dot{\langle a_2 \rangle} = -(\kappa_2' + i\Delta_\mathrm{c}) \langle a_2 \rangle - iv_2 \langle b \rangle - ig_2 \langle \sigma_2^- \rangle , \tag{7}$$

$$\dot{\langle b \rangle} = -(\kappa_b + i\Delta_\mathrm{c}) \langle b \rangle - iv_1^* \langle a_1 \rangle - iv_2^* \langle a_2 \rangle , \tag{8}$$

$$\dot{\langle \sigma_1^- \rangle} = -(\gamma_\perp + i\Delta_\mathrm{a}) \langle \sigma_1^- \rangle - ig_1^* \langle a_1 \rangle , \tag{9}$$

$$\dot{\langle \sigma_2^- \rangle} = -(\gamma_\perp + i\Delta_\mathrm{a}) \langle \sigma_2^- \rangle - ig_2^* \langle a_2 \rangle , \tag{10}$$

where $\kappa_{1,2}' = \kappa_{1,2} + \gamma_\mathrm{las}$, $\kappa_b = \kappa_{b,\mathrm{loss}} + \gamma_\mathrm{las}$, and $\gamma_\perp = \gamma_{\|}/2 + \gamma_\mathrm{las}$. Setting the time derivatives to zero, we find the general steady state solution for the amplitude of cavity 2 as

$$\langle a_2 \rangle_\mathrm{ss} = \frac{A}{B}, \tag{11}$$

where

$$A = -i\mathcal{E}_1 \left(\frac{v_2}{\kappa_b + i\Delta_\mathrm{c}} \right) \frac{v_1^*}{\kappa_1' + i\Delta_\mathrm{c} + \dfrac{|g_1|^2}{\gamma_\perp + i\Delta_\mathrm{a}} + \dfrac{|v_1|^2}{\kappa_b + i\Delta_\mathrm{c}}}, \tag{12}$$

and

$$B = -(\kappa_2' + i\Delta_\mathrm{c}) - \frac{|v_2|^2}{\kappa_b + i\Delta_\mathrm{c}} - \frac{|g_2|^2}{\gamma_\perp + i\Delta_\mathrm{a}} + \frac{|v_1 v_2|^2}{(\kappa_b + i\Delta_\mathrm{c})^2} \frac{1}{\kappa_1' + i\Delta_\mathrm{c} + \dfrac{|g_1|^2}{\gamma_\perp + i\Delta_\mathrm{a}} + \dfrac{|v_1|^2}{\kappa_b + i\Delta_\mathrm{c}}}. \tag{13}$$

Solutions for the steady state amplitudes of cavity 1 and fiber mode b then follow from

$$\langle a_1 \rangle_\mathrm{ss} = -\frac{i\mathcal{E}_1 + \dfrac{v_1 v_2^*}{\kappa_b + i\Delta_\mathrm{c}} \langle a_2 \rangle_\mathrm{ss}}{\kappa_1' + i\Delta_\mathrm{c} + \dfrac{|g_1|^2}{\gamma_\perp + i\Delta_\mathrm{a}} + \dfrac{|v_1|^2}{\kappa_b + i\Delta_\mathrm{c}}}, \tag{14}$$

and

$$\langle b \rangle_\mathrm{ss} = -\frac{iv_1^*}{\kappa_b + i\Delta_\mathrm{c}} \langle a_1 \rangle_\mathrm{ss} - \frac{iv_2^*}{\kappa_b + i\Delta_\mathrm{c}} \langle a_2 \rangle_\mathrm{ss} . \tag{15}$$

The output photon flux from cavity 2, in the linear regime, is then

$$|\langle a_{\mathrm{out},2} \rangle|^2 = 2\kappa_{2r} |\langle a_2 \rangle|^2 . \tag{16}$$

When plotting this quantity, we normalize by the on-resonance ($\Delta_\mathrm{c} = 0$) flux with no atoms ($g_{1,2} = 0$).

3. Normal mode description

We assume, for simplicity, that all of the parameters in the Hamiltonian are real. Considering just the cavities and coupling fiber, we can move to a normal mode picture, with normal mode operators given by

$$d = \frac{1}{\sqrt{2}\tilde{v}} (v_2 a_1 + v_1 a_2), \quad c_\pm = \frac{1}{2\tilde{v}} (v_1 a_1 + v_2 a_2) \pm \frac{1}{\sqrt{2}} b, \tag{17}$$

where

$$\tilde{v} = \sqrt{\frac{(v_1^2 + v_2^2)}{2}}. \tag{18}$$

Expressed in terms of these normal mode operators, the Hamiltonian becomes

$$\begin{aligned}H &= \Delta_{\rm c} d^\dagger d + \left(\Delta_{\rm c} + \sqrt{2}\tilde{v}\right) c_+^\dagger c_+ + \left(\Delta_{\rm c} - \sqrt{2}\tilde{v}\right) c_-^\dagger c_- + \Delta_{\rm a}\left(\sigma_1^+\sigma_1^- + \sigma_2^+\sigma_2^-\right) \\ &+ \mathcal{E}_{\rm p}\frac{v_1}{2\tilde{v}}\left(c_+^\dagger + c_+\right) + \mathcal{E}_{\rm p}\frac{v_1}{2\tilde{v}}\left(c_-^\dagger + c_-\right) + \mathcal{E}_{\rm p}\frac{v_2}{\sqrt{2}\tilde{v}}\left(d^\dagger + d\right) \\ &+ \frac{1}{2\tilde{v}}\left[\left(c_+^\dagger + c_-^\dagger\right)\left(v_1 g_1 \sigma_1^- + v_2 g_2 \sigma_2^-\right) + \text{H.c.}\right] + \frac{1}{\sqrt{2}\tilde{v}}\left[d^\dagger\left(v_2 g_1 \sigma_1^- + v_1 g_2 \sigma_2^-\right) + \text{H.c.}\right]. \end{aligned} \tag{19}$$

Let us now assume that the normal mode splitting $\sqrt{2}\tilde{v}$ is large compared to all other parameters. If the atomic transition frequency is close to the bare cavity resonance ($\omega_{\rm a} \simeq \omega_{\rm c}$), and the probe laser frequency is scanned just in this vicinity as well, then it is possible to focus on the system dynamics involving only the resonant mode d. That is, we can focus on the reduced Hamiltonian

$$\begin{aligned}H_d &= \Delta_{\rm c} d^\dagger d + \Delta_{\rm a}\left(\sigma_1^+\sigma_1^- + \sigma_2^+\sigma_2^-\right) + \mathcal{E}_{\rm p}\frac{v_2}{\sqrt{2}\tilde{v}}\left(d^\dagger + d\right) + \frac{1}{\sqrt{2}\tilde{v}}\left[d^\dagger\left(v_2 g_1 \sigma_1^- + v_1 g_2 \sigma_2^-\right) + \text{H.c.}\right] \\ &\equiv \Delta_{\rm c} d^\dagger d + \sum_{i=1,2}\left[\Delta_{\rm a}\sigma_i^+\sigma_i^- + g_{di}\left(d^\dagger\sigma_i^- + \sigma_i^+ d\right)\right] + \mathcal{E}_d\left(d^\dagger + d\right), \end{aligned} \tag{20}$$

where

$$g_{d1} = \frac{v_2}{\sqrt{2}\tilde{v}} g_1, \quad g_{d2} = \frac{v_1}{\sqrt{2}\tilde{v}} g_2, \quad \text{and} \quad \mathcal{E}_d = \mathcal{E}_{\rm p}\frac{v_2}{\sqrt{2}\tilde{v}}. \tag{21}$$

Now considering the dissipative terms in the master equation and noting the inverse relations

$$b = \frac{1}{\sqrt{2}}\left(c_+ - c_-\right), \quad a_1 = \frac{1}{2\tilde{v}}\left[v_1\left(c_+ + c_-\right) + \sqrt{2}v_2 d\right], \quad a_2 = \frac{1}{2\tilde{v}}\left[v_2\left(c_+ + c_-\right) - \sqrt{2}v_1 d\right], \tag{22}$$

one can see that, in general, the decay of each normal mode cannot be decoupled from the other normal modes, i.e., there are cross-terms between the normal mode operators arising from the Lindblad forms $\kappa_1\mathcal{D}[a_1]\rho + \kappa_2\mathcal{D}[a_2]\rho + \kappa_{b,\rm loss}\mathcal{D}[b]\rho$ (for simplicity, we set $\gamma_{\rm las} = 0$ and ignore phase damping in this discussion). Once again, however, if $\sqrt{2}\tilde{v}$ is sufficiently large, then the cross-terms can be neglected (as in a rotating-wave approximation) and the normal modes can be regarded as decaying according to

$$\kappa_d\mathcal{D}[d]\rho + \kappa_+\mathcal{D}[c_+]\rho + \kappa_-\mathcal{D}[c_-]\rho, \tag{23}$$

with

$$\kappa_d = \frac{v_2^2\kappa_1 + v_1^2\kappa_2}{2\tilde{v}^2} \quad \text{and} \quad \kappa_+ = \kappa_- = \frac{1}{2}\left(\kappa_{b,\rm loss} + \frac{v_1^2\kappa_1 + v_2^2\kappa_2}{2\tilde{v}^2}\right). \tag{24}$$

So, we can in turn focus on the reduced master equation,

$$\dot{\rho} = -i[H_d, \rho] + \kappa_d\mathcal{D}[d]\rho + \gamma_\perp\left(\mathcal{D}[\sigma_1^-]\rho + \mathcal{D}[\sigma_2^-]\rho\right), \tag{25}$$

for probe driving around the atom-cavity resonance $\omega_{\rm c} \simeq \omega_{\rm a}$ and far from the normal modes at $\omega_{\rm c} \pm \sqrt{2}\tilde{v}$.

4. Vacuum Rabi splitting of the fiber-dark mode d

The photon flux transmitted through the coupled-cavities system is determined by the output photon flux from cavity 2, i.e., by $2\kappa_{2r}\langle a_2^\dagger a_2\rangle$. Given weak probe driving strength in supplementary equation (25), and the relationship between a_2 and d in supplementary equation (22), the transmitted spectrum will show, for sufficiently large atom-cavity coupling strengths, vacuum Rabi splitting of the fiber-dark mode d given by

$$\pm\sqrt{(g_{d1}^2 + g_{d2}^2) - \frac{1}{4}(\kappa_d - \gamma_\perp)^2}, \tag{26}$$

which is approximately $\pm\sqrt{g_{d1}^2 + g_{d2}^2}$ for the parameters of the experiment.

5. *Saturation*

From the semiclassical (nonlinear) equations of motion in steady state, we can eliminate the atomic variables, as well as the connecting-fiber mode amplitude, to obtain the following coupled equations for the amplitudes of the two cavity modes,

$$-i\frac{\mathcal{E}_{\rm p}}{\kappa_1'} = \langle a_1\rangle \left\{ \left(1 + i\frac{\Delta_{\rm c}}{\kappa_1'} + \frac{v_1^2/\kappa_1'}{\kappa_b + i\Delta_{\rm c}}\right) + \left(1 - i\frac{\Delta_{\rm a}}{\gamma_\perp}\right) \sum_{j_1} \frac{C_{1j_1}}{1 + \frac{\Delta_{\rm a}^2}{\gamma_\perp^2} + \frac{|\langle a_1\rangle|^2}{n_{1j_1}}} \right\} + \frac{v_1 v_2/\kappa_1'}{\kappa_b + i\Delta_{\rm c}} \langle a_2\rangle\,, \tag{27}$$

$$0 = \langle a_2\rangle \left\{ \left(1 + i\frac{\Delta_{\rm c}}{\kappa_2'} + \frac{v_2^2/\kappa_2'}{\kappa_b + i\Delta_{\rm c}}\right) + \left(1 - i\frac{\Delta_{\rm a}}{\gamma_\perp}\right) \sum_{j_2} \frac{C_{2j_2}}{1 + \frac{\Delta_{\rm a}^2}{\gamma_\perp^2} + \frac{|\langle a_2\rangle|^2}{n_{2j_2}}} \right\} + \frac{v_1 v_2/\kappa_2'}{\kappa_b + i\Delta_{\rm c}} \langle a_1\rangle\,, \tag{28}$$

where

$$C_{lj_l} = \frac{g_{lj_l}^2}{\kappa_l'\gamma_\perp} \quad \text{and} \quad n_{lj_l} = \frac{\gamma_\perp\gamma_\parallel}{4g_{lj_l}^2} \qquad (l = 1, 2). \tag{29}$$

Here, g_{lj_l} denotes the coupling strength of atom j_l to cavity mode l. Note that we will assume for simplicity that the parameters $\{g_{1j_1}, g_{2j_2}\}$, $\{v_1, v_2\}$, and $\mathcal{E}_{\rm p}$ are all real. In what follows, we will also assume resonance between atoms, field modes and driving laser field, i.e., $\Delta_{\rm c} = \Delta_{\rm a} = 0$.

a. *Atoms in cavity 1 only*

Setting $C_{2j_2} = 0$ (no atoms in cavity 2), we can eliminate the amplitude $\langle a_2\rangle$ to obtain an equation for the amplitude $\langle a_1\rangle$ alone, which in scaled variables takes the form

$$-iy_1 = X_1 \left\{ 1 + \frac{v_1^2/(\kappa_b\kappa_1')}{1 + v_2^2/(\kappa_b\kappa_2')} + C_{1,(0)} \sum_{j_1} \frac{C_{1j_1}/C_{1,(0)}}{1 + |X_1|^2 \left(g_{1j_1}/g_{1,(0)}\right)^2} \right\}, \tag{30}$$

where

$$y_1 = \frac{\mathcal{E}_{\rm p}}{\kappa_1'\sqrt{n_{1,\rm sat}}}, \quad X_1 = \frac{\langle a_1\rangle}{\sqrt{n_{1,\rm sat}}}, \quad n_{1,\rm sat} = \frac{\gamma_\perp\gamma_\parallel}{4g_{1,(0)}^2}, \quad C_{1,(0)} = \frac{g_{1,(0)}^2}{\kappa_1'\gamma_\perp}. \tag{31}$$

Here, $g_{1,(0)}$ is the single-atom coupling strength for an atom located at a potential minimum of the dipole trap in cavity 1. The normalized probe transmission through the system (i.e., the normalized output intensity from cavity 2) in the semiclassical model is given by

$$T = \left|\frac{\langle a_2\rangle}{\langle a_2\rangle_0}\right|^2 = \frac{1}{y_1^2}\left(1 + \frac{v_1^2}{\kappa_b\kappa_1'} + \frac{v_2^2}{\kappa_b\kappa_2'}\right)^2 \frac{|X_1|^2}{\left(1 + \frac{v_2^2}{\kappa_b\kappa_2'}\right)^2}, \tag{32}$$

where $\langle a_2\rangle_0$ is the mode amplitude of cavity 2 for resonant driving and no atoms in either cavity. Finally, the input probe power can be related to y_1 and $n_{1,\rm sat}$ through the relation

$$P_{\rm in} = \frac{\mathcal{E}_{\rm p}^2}{2\kappa_{1l}}\hbar\omega_{\rm p} = \frac{\mathcal{E}_{\rm p}^2}{2\kappa_{1l}}\frac{2\pi\hbar c}{\lambda} = y_1^2\left(\frac{2\pi\hbar c}{\lambda}\right)\left(\frac{{\kappa_1'}^2}{2\kappa_{1l}}\right) n_{1,\rm sat}. \tag{33}$$

To compare theory and experiment, we plot T versus $P_{\rm in}$ as given in supplementary equation (33), with $n_{1,\rm sat}$ as a scaling factor.

b. Atoms in cavity 2 only

Now setting $C_{1j_2} = 1$ (no atoms in cavity 1), we can similarly eliminate the amplitude $\langle a_1 \rangle$ to obtain an equation for the amplitude $\langle a_2 \rangle$ alone, which in scaled variables takes the form

$$iy_2 = X_2 \frac{v_1 \kappa_2'}{v_2 \kappa_1'} \left(1 + \frac{\kappa_b \kappa_1'}{v_1^2} \right) \left\{ 1 + \frac{v_2^2 \kappa_1'}{v_1^2 \kappa_2'} \frac{1}{1 + \kappa_b \kappa_1'/v_1^2} + C_{2,(0)} \sum_{j_2} \frac{C_{2j_2}/C_{2,(0)}}{1 + |X_2|^2 \left(g_{2j_2}/g_{2,(0)} \right)^2} \right\}, \tag{34}$$

where

$$y_2 = \frac{\mathcal{E}_{\rm p}}{\kappa_1' \sqrt{n_{2,\rm sat}}}, \quad X_2 = \frac{\langle a_2 \rangle}{\sqrt{n_{2,\rm sat}}}, \quad n_{2,\rm sat} = \frac{\gamma_\perp \gamma_\parallel}{4 g_{2,(0)}^2}, \quad C_{2,(0)} = \frac{g_{2,(0)}^2}{\kappa_2' \gamma_\perp}. \tag{35}$$

Here, $g_{2,(0)}$ is the single-atom coupling strength for an atom located at a potential minimum of the dipole trap in cavity 2. The normalized probe transmission through the system in this case is given by

$$T = \left| \frac{\langle a_2 \rangle}{\langle a_2 \rangle_0} \right|^2 = \frac{1}{y_2^2} \left(\frac{1 + v_1^2/(\kappa_b \kappa_1') + v_2^2/(\kappa_b \kappa_2')}{v_1 v_2/(\kappa_b \kappa_2')} \right)^2 |X_2|^2 , \tag{36}$$

and the relationship to the input power becomes

$$P_{\rm in} = \frac{\mathcal{E}_{\rm p}^2}{2\kappa_{1l}} \hbar \omega_{\rm p} = y_2^2 \left(\frac{2\pi \hbar c}{\lambda} \right) \left(\frac{{\kappa_1'}^2}{2\kappa_{1l}} \right) n_{2,\rm sat}. \tag{37}$$

c. Position dependence of the atom-cavity coupling

From [2], we take the (square of the) atom-cavity mode coupling strength, in both cavities, to have the form ($l = 1, 2$)

$$\begin{aligned} g_l(r,\phi,z)^2 = {} & \mathcal{C} \left(\frac{\beta}{2q} \right)^2 \left\{ \left[(1-s) K_0(qr) + (1+s) K_2(qr) \cos(2\phi) \right]^2 + (1+s)^2 K_2^2(qr) \sin^2(2\phi) \right\} \cos^2(\beta z) \\ & + \mathcal{C} \left\{ K_1^2(qr) \cos^2(\phi) \right\} \sin^2(\beta z), \end{aligned} \tag{38}$$

where $\mathcal{C}$ is a constant and we use cylindrical coordinates, with the z-direction along the fiber axis and r ($> a$) measured from the center of the fiber. The propagation constant $\beta = 7.87925 \times 10^6$ m^{-1}, and $q = \sqrt{\beta^2 - n_2^2 k^2}$ with $n_2 = 1$ and $k = 2\pi/\lambda$ ($\lambda = 852$ nm). The parameter $s = -0.828$ depends on q, on $h = \sqrt{k^2 n_1^2 - \beta^2}$ ($n_1 = 1.4525$), and on the radius (a) of the nanofiber [2]. With respect to the position of a minimum of the dipole trapping potential located at ($r = r_{l,0}, \phi = 0, z = 0$), we find that the above function is in fact very well approximated by the simplified form

$$g_l(r,\phi,z)^2 = g_{l,(0)}^2 \frac{1}{2} \left[1 + A + B \cos(2\beta z) \right] \frac{e^{-2q'(r - r_{l,0})}}{r/r_{l,0}} \cos^2(\phi), \tag{39}$$

with $A + B = 1$ and $q' \simeq 1.3q$. We shall use this approximate form in the calculations that follow.

d. Integral approximation to the summation over atoms

Assuming sufficiently many atoms in each cavity, we replace the summations in (30) and (34) as follows:

$$C_{l,(0)} \sum_{j_l} \frac{C_{lj_l}/C_{l,(0)}}{1 + |X_l|^2 \left(g_{lj_l}/g_{l,(0)} \right)^2} \longrightarrow C_{l,(0)} \int_0^L \int_{-\pi}^{\pi} \int_a^\infty \rho_l(r,\phi,z) \frac{\left(g_l(r,\phi,z)/g_{l,(0)} \right)^2}{1 + |X_l|^2 \left(g_l(r,\phi,z)/g_{l,(0)} \right)^2} \, r dr d\phi dz, \tag{40}$$

where $\rho_l(r,\phi,z)$ is the atom density distribution in cavity l. With respect to the z-direction, atoms are tightly confined by a standing wave optical potential. However, this standing wave is incommensurate with the cavity mode standing wave and so, on average, the atomic distribution along the z-direction, with regards to the cavity mode, can be regarded as uniform. That is, we take $\rho_l(r,\phi,z) = \rho_l(r,\phi)$, and then the integration over z can be carried out exactly, reducing the above expression to

$$C_{l,(0)} L \frac{1}{|X_l|^2} \int_{-\pi}^{\pi} \int_a^\infty \rho_l(r,\phi) f_l(r,\phi) \, r dr d\phi, \tag{41}$$

where

$$f_l(r,\phi) = 1 - \left[\left(1 + A\left|X_l\right|^2 \frac{e^{-2q'(r-r_0)}}{r/r_0}\cos^2(\phi)\right)\left(1 + \left|X_l\right|^2 \frac{e^{-2q'(r-r_0)}}{r/r_0}\cos^2(\phi)\right)\right]^{-1/2}. \tag{42}$$

Making a harmonic approximation to the atom trapping potential, the atomic density distribution can be written, in Cartesian coordinates, as

$$\rho_l(x,y) = \rho_{l,0} e^{-(x-x_{l,0})^2/\sigma_{l,x}^2} e^{-y^2/\sigma_{l,y}^2}, \tag{43}$$

where $x_{l,0} = r_{l,0}$ and $\sigma_{x,y} = \sqrt{2k_{\rm B}T/m\omega_{x,y}^2}$ for a gas at temperature T and with trapping frequencies $\omega_{x,y}$.

The double integral (41) can be rewritten in Cartesian coordinates as

$$C_{l,(0)} L\rho_{l,0} \frac{1}{\left|X_l\right|^2} \iint_{x^2+y^2\geq a^2} e^{-(x-x_{l,0})^2/\sigma_{l,x}^2} e^{-y^2/\sigma_{l,y}^2} f_l(x,y)\, dxdy. \tag{44}$$

In the x-direction, the function $f_l(x,y)$ varies slowly (i.e., approximately linearly) in comparison to the Gaussian density distribution for the characteristic parameters of the experiment. Given this, and assuming $\sigma_{l,x} \ll x_{l,0} - a$ ($x_{l,0} - a$ is the distance from the trap center to the surface of the nanofiber), the integral is well approximated by

$$C_{l,(0)} L\rho_{l,0} \frac{1}{\left|X_l\right|^2} \sqrt{\pi}\sigma_{l,x}\sigma_{l,y} \int_{-\infty}^{\infty} e^{-u^2} f_l(x_{l,0},u)\, du, \tag{45}$$

where

$$f_l(x_{l,0},u) = 1 - \frac{1}{\sqrt{\left(1 + A\left|X_l\right|^2 s(x_{l,0},u)\right)\left(1 + \left|X_l\right|^2 s(x_{l,0},u)\right)}}, \tag{46}$$

with

$$s(x_{l,0},u) = \frac{\exp\left[-2q' x_{l,0}\left(\sqrt{1 + (\sigma_{l,y}^2/x_{l,0}^2)u^2} - 1\right)\right]}{\left[1 + (\sigma_{l,y}^2/x_{l,0}^2)u^2\right]^{3/2}}. \tag{47}$$

The trapping along the y-direction is typically much weaker than in the other directions (i.e., $\sigma_{l,y} \gtrsim 5\sigma_{l,x}$), but, given uncertainty in the exact trapping parameters, and making the (not unreasonable) assumption that $\sigma_{l,y}^2/x_{l,0}^2 \ll 1$, we can set $s(x_{l,0},u) = 1$ in the integral, and then (45) reduces to

$$C_{l,(0)} N_{l,\rm eff} \frac{2}{(1+A)} \frac{1}{\left|X_l\right|^2} \left[1 - \frac{1}{\sqrt{(1 + A\left|X_l\right|^2)(1 + \left|X_l\right|^2)}}\right], \tag{48}$$

where $N_{l,\rm eff} = \rho_{l,0}(L/2)(1+A)\pi\sigma_{l,x}\sigma_{l,y}$. In our comparison between theory and experiment, we use (48) to approximate the summations in (30) and (34). We use a value $A = 0.17$, deduced from the experimental parameters and (38). As mentioned earlier, matching the theory curves for T versus $P_{\rm in}$ to the experimental data yields estimates of $n_{l,\rm sat}$, from which we can deduce values for $g_{l,(0)}$. From these, and the estimates of $g_{l,\rm eff}$ obtained from the weak-field transmission spectra, we then deduce estimates for $N_{l,\rm eff}$.

Supplementary Note 2: Experimental Setup

The schematic of the experimental setup is shown in supplementary Fig. 2. Each cavity consists of a nanofiber, both ends of which are connected to standard single-mode fibers through tapered regions, and sandwiched by a pair of fiber-Bragg-grating (FBG) mirrors[1]. The single-pass loss inside each cavity is 2%, which is dominated by the losses in two fiber splices. The diameter and length of the nanofibers are 400 nm and 3 mm, respectively. The nanofibers are located in two separate UHV chambers.

We put three-paddle polarization controllers in the connecting fiber and cavity 2 to compensate polarization rotation in the optical fiber. For the case of $L_f = 0.83$ m (Figs. 3a and c in the main text), we replace the polarization controller in the connecting fiber with a two-paddle one because of the limitation of the fiber length, which cause the imperfect polarization compensation. The lasers for the optical trap and probe are mixed by using dichroic mirrors and coupled to the optical fiber. In contrast, the optical pumping pulses are irradiated on the nanofiber from its side. The polarizations of the trapping fields are linearly polarized and parallel to each other at the nanofiber regions. The transmitted probe beam passes the filtering system to block unwanted stray light, and is detected by an avalanche photodetector. The detection efficiency including the transmittance of the filtering system and the quantum efficiency of the detector is 0.26.

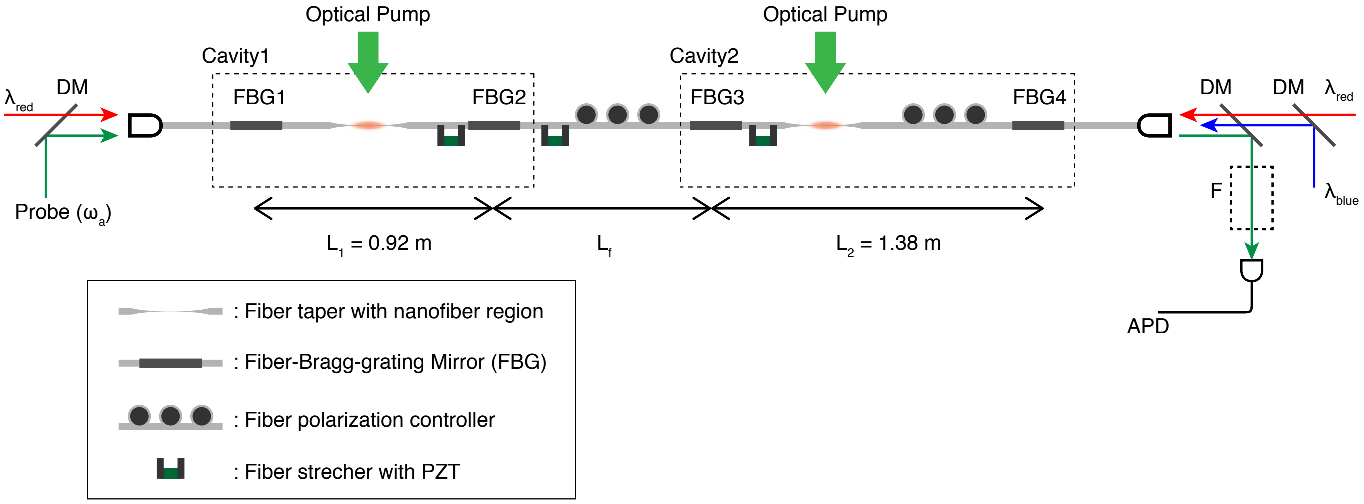

Supplementary Fig. 2. Schematic of the setup. Cavity 1 of length $L_1 = 0.92$ m and Cavity 2 of length $L_2 = 1.38$ m are connected by a fiber of length L_f. DM: Dichroic mirror, F: filters, APD: avalanche photodiode.

A. Measurement sequence and data analysis

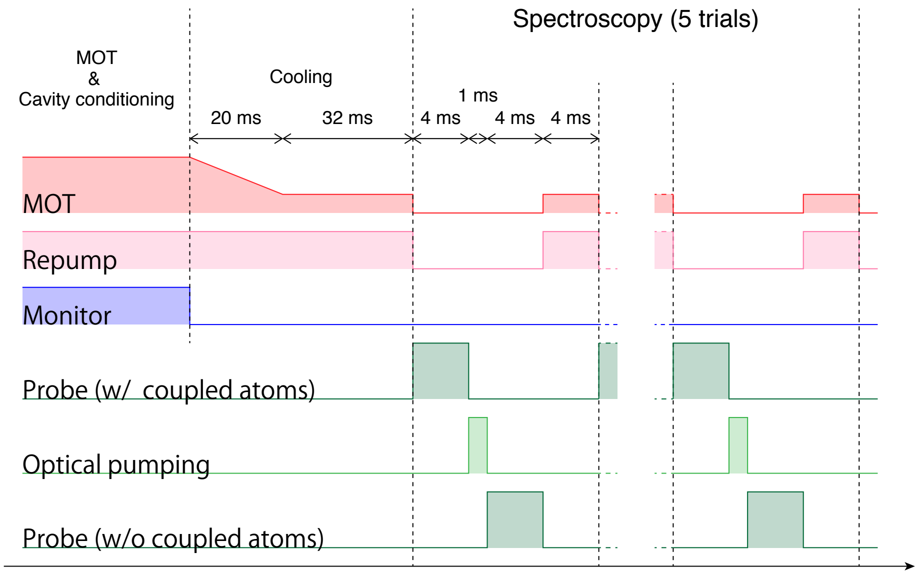

Supplementary Fig. 3. Sequence diagram of the spectroscopy. After the preparation of atoms in the MOT and additional cooling, the spectroscopy procedure repeats five times.

The schematic diagram of the pulse sequence for the transmission spectroscopy is shown in Fig. 3. Each sequence starts from cooling and trapping cesium atoms in the two UHV chambers by using standard six-beam magneto-optical traps (MOT). We use the D_2 line F=4→F'=5 transition for cooling and the F=3→F'=4 transition for repumping in the MOTs. In the loading stage of the MOTs, the total intensity and the detuning of the cooling beams are, respectively, $16I_s$ and -1.25Γ, where I_s and Γ are the saturation intensity and natural linewidth of the cooling transition.

The number of the atoms in the MOTs saturates within the loading time of about 500 ms. After the preparation of the atoms in the MOT, we adjust the resonance frequency of the cavity array close to the F=4→F'=5 transition. We input a monitor beam with the power of 130 pW and the frequency locked to the F=4→F'=5 transition, while changing the cavity lengths of the cavity array (cavity 1, 2, and the connecting fiber), and the transmission of the monitor beam is used as a start trigger for the following sequence. The MOTs are kept during the monitoring stage, and the positions have a small offset from the nanofibers to keep the cavities without atoms. When the transmission surpass a threshold, we switch off the beam for the monitor, and change the detuning and intensity of the cooling laser to $0.5I_s$ and -3.45Γ, respectively, and hold for 32 ms for further cooling and loading the atoms into the optical trap. During the cooling stage, the positions of the atoms are overlapped onto the nanofibers to load them in the traps. After the loading atoms into the traps, we send a probe pulse and sweep the frequency detuning to the F=4→F'=5 over ±30 MHz within 4 ms. To observe the transmission spectrum without atoms (empty cavity), we optically pump the atoms into the F=3 state, which is off-resonant to the cavity field, by using the optical pumping pulse irradiated from the side of nanofibers. We use the F=4→F'=3 transition for the pumping, and the pulse duration is 1 ms. After the optical pumping, we send a probe pulse again, and sweep the frequency detuning in the same manner as the previous probe pulse. We then switch on the cooling and repumping lasers to cool and load the atoms into the optical trap again. The cooling duration is 4 ms. The spectroscopy procedure repeats five times per single MOT loading event. Figure 4 shows typical transmission spectra for a sequence of five spectroscopy procedures, each of which is an average of about 15 sets of data. It can be seen that the splittings for the center peak (fiber-dark mode) reduces as the spectroscopy procedure is repeated, which is due to the decrease of the number of the atoms in the optical trap. We use the average of the second, third, and forth spectra (after taking an average of about 15 sets of data for each of the five spectroscopy procedures in the same way as the spectra in Fig. 4) for the data analysis in the main text.

In the data analysis, the observed spectrum without the atom-cavity interaction (empty cavity), where the atoms are optically pumped into the F=3 state, is used to judge whether the cavity array is resonant to the atomic transition or not. When all cavities (Cavity1, 2 and the channel part) are resonant to the atom, the transmission spectrum has a symmetric triplet peaks centered at the zero atom-probe detuning (see Figs 2a, 3a, 3c in the main text). In order to choose the data in the relevant condition, we put the following criteria to filter the data; the spectrum of the empty cavity has a largest transmission peak around the atomic resonance frequency within ±0.9 MHz, and has symmetrical sideband peaks at $\pm\sqrt{2}\tilde{v}$ within ±1.8 MHz.

SUPPLEMENTARY REFERENCES

[1] Shinya Kato and Takao Aoki, Strong Coupling between a Trapped Single Atom and an All-Fiber Cavity, Phys. Rev. Lett. **115** 093603 (2015).

[2] C. Lacroûte,, K. S. Choi, A. Goban, D. J. Alton, D. Ding, N. P. Stern, and H. J. Kimble, A state-insensitive, compensated nanofiber trap, New J. Phys. **14**, 023056 (2012).

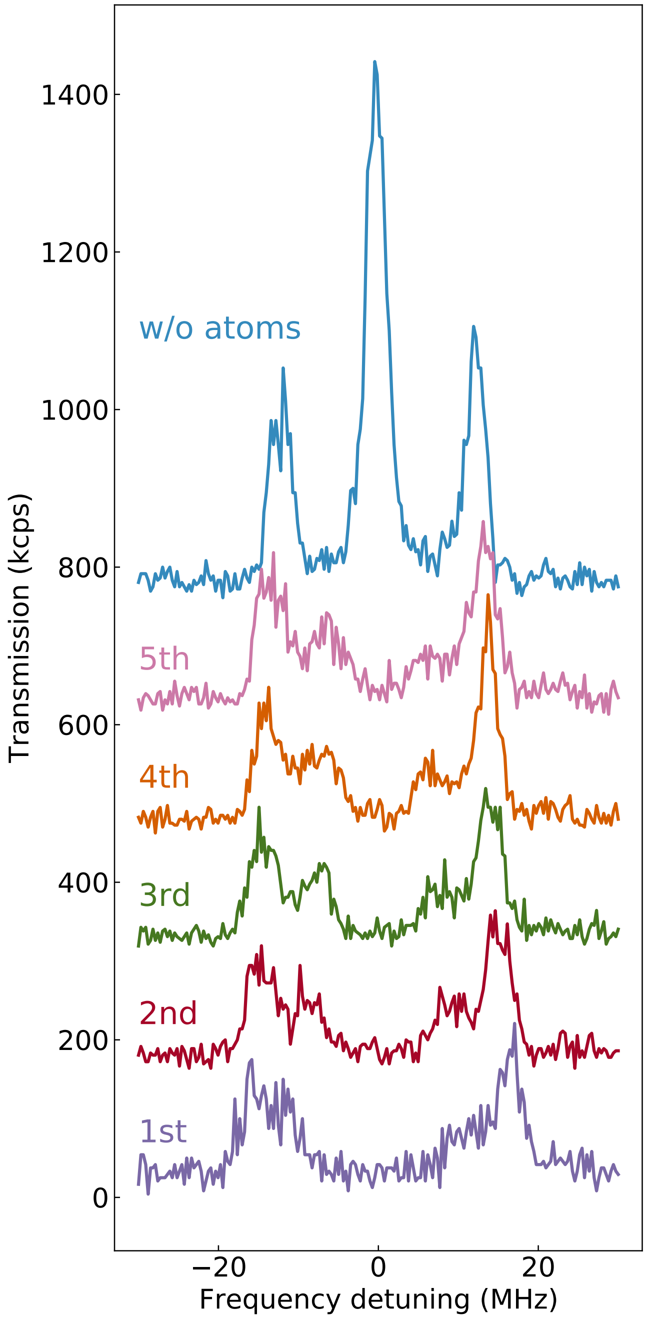

Supplementary Fig. 4. Typical transmission spectra for the five consecutive spectroscopy procedures per single MOT loading event. The experimental condition is the same as that of Fig. 2d in the main text. The spectra include vertical offsets for clarity.
