# Supplementary material for: Observation of dressed states of distant atoms with delocalized photons in coupled-cavities quantum electrodynamics
Source: Nat Commun. 2019 Mar 11;10:1160. doi: 10.1038/s41467-019-08975-8 (PMC6411748; doi:10.1038/s41467-019-08975-8)
Supplement: Supplementary file 2 — Peer Review [file 41467_2019_8975_MOESM2_ESM.pdf]

Reviewers' comments:

Reviewer #1 (Remarks to the Author):

In the manuscript "Coupled-cavities quantum electrodynamics: observation of dressed states of distant atoms with delocalized photons", Kato et al. perform experiments with a fiber-integrated cavity quantum electrodynamics (cQED) set-up, in which two optical nanofiber-based Fabry-Perot resonators are coupled by a meter-long fiber segment. Two atomic ensembles are optically trapped in the evanescent field surrounding the optical nanofibers. Recording transmission spectra without, with one, and with two atomic ensembles, they show collective strong coupling between the atomic ensembles and the normal mode of the coupled cavities for which the light predominantly resides in the cavities (i.e., for which the fiber that connects the two cavities is dark).

The paper presents an interesting experimental system that the authors master very well. To my knowledge, this is the first time that two atomic ensembles, separated by a meter-scale distance, are optically interfaced and coherently coupled by means of an optical fiber. This clearly demonstrates the potential of the approach, which realizes a promising platform for quantum simulation and quantum information processing. Moreover, the data as well as the theoretical modelling / analysis are convincing and sound and the manuscript in very well written and accessible to a broader audience. I am therefore in favor of publication of this work in Nature Communications.

Prior to publication, the authors should address the following points:

- Currently, many atoms (~40-100) are loaded in each cavity. This allows the authors to increase the effective atom-cavity coupling strength by an order of magnitude and, thus, to make their scheme work for the parameters of their photonic system. At the same time, however, the resulting system dynamics is classical and linear at the single-photon level. In this context, the authors write: "By reducing the number of atoms in each cavity to one [27], on the other hand, it will be possible to construct a fiber network of single-atom cavity QED systems." Here, Ref. [27] refers to former experimental work from the same group. For the reader, it would be instructive to learn about the challenges that one faces in this endeavor. Would it be possible to simply connect two cavities from Ref. [27] and make the scheme work with a single atom in each cavity? If not, the authors should comment on the prospects of implementing the required improvements.

- Currently, the authors probe the system by recording the transmission through the two coupled cavities and the resulting transmission signal seems to be independent of whether two atomic ensembles with N atoms each are coupled to the two cavities or one atomic ensemble with 2N atoms is coupled to only one of the two cavities. I agree with the authors' reasoning that the consistency between the three observed Rabi-splittings (atoms loaded only in cavity 1 & 2, respectively, and atoms loaded in both cavities) is a sufficient signature of the collective dressed states of distant atoms with delocalized photons of the fiber-dark mode. Still, it would be interesting to learn whether the system features characteristics which are directly related to the delocalization of the photons, e.g., due to retardation. If so, the authors should highlight these features and comment on how they could be probed.

- In the summary, it is stated that "the interaction of atoms with the fiber-dark mode can be utilized in quantum gates for distributed quantum computation that is insensitive to fiber losses." Here, it would be helpful if the authors were more specific concerning the conditions under which fiber losses do not play a role. It seems to me that the fiber-dark mode is no longer perfectly dark if the fiber connecting the two cavities features propagation loss (consider the extreme case when the connecting fiber is cut in half). Thus, the insensitivity to fiber loss only seems to hold in a limiting case.

Reviewer #2 (Remarks to the Author):

The paper by S. Kato et al. reports on the initial observation of dressed states of distant atoms via coupled- CQED systems. Thanks to the use of all-fibered elements – fiber coupled atoms, Bragg mirrors and fiber to connect the two cavities – the authors have for the first time succeeded in showing a coherent reversible coupling between atoms separated by more than a meter. Each experimental steps are detailed and carefully characterized. Experimental methods and theoretical analysis are both very solid and I do not have any comments on them.

The demonstrated capability is a long-awaited and significant milestone in the field of quantum electrodynamics, including for quantum computing and communication. It will reach a broad readership, as CQED is pursued in a variety of physical systems. The all-fibered features demonstrated here pave the way to complex topologies. I therefore recommend the publication of this study in Nature Communications after the authors address the following points.

-I found the introduction not very well adapted to a broad audience. Potential applications are only described very shortly. An extended paragraph about future prospects opened by this capability would be interesting in the introduction.

-The authors could better emphasize what made this achievement possible. How the losses for instance are critical to observe this feature? Would have it been possible with current free-space cqed systems ?

- The authors use a relatively short fiber. I wonder what would be the effect of a much longer fiber, with a "significant" delay between the two cavities?

- At the end of the paper, saturation behaviors are given and fitted. The authors then give different quantities such as N_eff or n_sat without further details (sending then the reader to the SI). For the self-consistency of the main text, a few sentences to give the physical meaning will be welcome here.

- In the conclusion, the authors discuss the prospects. What should be the required improvements in their system (coupling, loss,...) to be able to tackle some proposals (entangling or swapping gates) as proposed in the seminal reference [15] ?

- Captions of the figures should be extended to be more self-consistent. For figure 1, the setup can be described. Captions of Figure 2 and 3 should include some comments to indicate to the readers what should be looked at the first sight. It will be a helpful guideline.

- The Methods section is limited to the preparation of the atoms, without discussing the dipole trapping. This discussion is given in section B of the SI. I believe this section should better appear in the Methods section for the completeness of the main text.

We thank both reviewers for their effort and valuable comments. In the following we reply to each of the comments raised by the reviewers, and describe the corresponding changes we made to the manuscript.

Reviewer #1:

We thank Reviewer #1 for expressing his/her view that our work "clearly demonstrates the potential of the approach, which realizes a promising platform for quantum simulation and quantum information processing," for his/her valuable comments and suggestions, and for recommending publication in Nature Communications. In the following, we reply to each of his/her comments.

- Currently, many atoms (~40-100) are loaded in each cavity. This allows the authors to increase the effective atom-cavity coupling strength by an order of magnitude and, thus, to make their scheme work for the parameters of their photonic system. At the same time, however, the resulting system dynamics is classical and linear at the single-photon level. In this context, the authors write: "By reducing the number of atoms in each cavity to one [27], on the other hand, it will be possible to construct a fiber network of single-atom cavity QED systems." Here, Ref. [27] refers to former experimental work from the same group. For the reader, it would be instructive to learn about the challenges that one faces in this endeavor. Would it be possible to simply connect two cavities from Ref. [27] and make the scheme work with a single atom in each cavity? If not, the authors should comment on the prospects of implementing the required improvements.

In principle, it is indeed possible to simply connect two cavities from Ref. [27] to construct a coupled-cavities QED system. However, there are two major technical improvements required to observe the vacuum Rabi splitting between a single atom and the fiber-dark mode. In Ref. [27], strong coupling between a single atom and a nanofiber cavity was demonstrated in the critical coupling regime for the cavity. On the other hand, in the present case, all three cavities have to be in the regime of over-coupling with respect to the inner mirrors, which results in the smaller ratio of g/κ compared to the case of Ref. [27]. Furthermore, the single-atom coupling

strength for the fiber-dark mode is reduced from that for a single cavity according to Eq. (7), which again results in smaller ratios of g/κ and g/γ compared to the case of Ref. [27]. Therefore, we need to further reduce the intrinsic losses (α_1, α_2, α_f) from the current values to achieve strong coupling between a single atom and the fiber-dark mode. Secondly, in order to accumulate a sufficient amount of data within a realistic time, we need to actively stabilize the three cavities to the atomic frequency and/or to extend the lifetime of the atom trap, since single atoms are loaded probabilistically. In contrast, ensembles of many atoms in the current study can be loaded deterministically. We do consider that demonstrating a coupled-cavities QED system with a single atom will be of significance, and we are making the above technical improvements toward this goal. Accordingly, we have added the following sentences in the summary paragraph of the main text: "In order to extend our work to the construction of a fiber network of coherently coupled single-atom cavity QED systems, we are currently making technical improvements to the setup, *e.g.*, further reduction of the internal losses in the cavities, active stabilization of the three cavities, and extending the lifetime of the atomic trap [38]."

- Currently, the authors probe the system by recording the transmission through the two coupled cavities and the resulting transmission signal seems to be independent of whether two atomic ensembles with N atoms each are coupled to the two cavities or one atomic ensemble with 2N atoms is coupled to only one of the two cavities. I agree with the authors' reasoning that the consistency between the three observed Rabi-splittings (atoms loaded only in cavity 1 & 2, respectively, and atoms loaded in both cavities) is a sufficient signature of the collective dressed states of distant atoms with delocalized photons of the fiber-dark mode. Still, it would be interesting to learn whether the system features characteristics which are directly related to the delocalization of the photons, e.g., due to retardation. If so, the authors should highlight these features and comment on how they could be probed.

It would indeed be interesting to further investigate the delocalization of photons for the fiber-dark mode. A straightforward extension of the present study would be

to drive and/or detect photons in the connecting fiber. The fiber-dark mode cannot be either driven or detected in this configuration. This would be more direct confirmation of the "fiber-dark" characteristics. It would also be interesting to investigate the transient dynamics of the system, where retardation may play an important role as Reviewer #1 suggests. In such experiments, it would also be possible to observe deterministic, reversible exchange of excitation between distant atoms. Accordingly, we have added the following sentences in the summary paragraph of the main text: "We note that the delocalization of photons for the fiber-dark mode would be explicitly demonstrated by the inability to drive or detect this mode through the connecting fiber, which could be facilitated by inserting a fiber beam splitter in the connecting fiber. Also, while in the present study the coherent coupling between distant atoms has been observed in the steady-state spectra, it will be possible, and very interesting, to also investigate transient dynamics of this system, where deterministic, reversible exchange of excitation between distant atoms would be observable."

- In the summary, it is stated that "the interaction of atoms with the fiber-dark mode can be utilized in quantum gates for distributed quantum computation that is insensitive to fiber losses." Here, it would be helpful if the authors were more specific concerning the conditions under which fiber losses do not play a role. It seems to me that the fiber-dark mode is no longer perfectly dark if the fiber connecting the two cavities features propagation loss (consider the extreme case when the connecting fiber is cut in half). Thus, the insensitivity to fiber loss only seems to hold in a limiting case.

We thank Reviewer #1 for pointing this out. The normal mode description including the fiber-dark mode is only valid when the coherent coupling rate between each cavity and the fiber is larger than the loss rates (*i.e.*, when the triplet peaks in Fig. 1(a) are spectrally resolved). We have modified the corresponding sentence as follows: "Furthermore, the interaction of atoms with the fiber-dark mode can be utilized in quantum gates for distributed quantum computation that is insensitive to fiber losses[15] (as long as the normal mode description is valid, *i.e.*, the coherent coupling rate between each cavity and the fiber is larger than the loss rates)."

Reviewer #2:

We thank Reviewer #2 for expressing his/her view that "the demonstrated capability is a long-awaited and significant milestone in the field of quantum electrodynamics, including for quantum computing and communication," for his/her valuable comments and suggestions, and for recommending publication in Nature Communications. In the following, we reply to each of his/her comments.

-I found the introduction not very well adapted to a broad audience. Potential applications are only described very shortly. An extended paragraph about future prospects opened by this capability would be interesting in the introduction.

We thank Reviewer #2 for this suggestion. We have extended the last paragraph of the introduction: "Our achievement is an important step towards the physical implementation of cavity QED-based distributed quantum computation[15-18] and a quantum network [19,20], where a large number of cavity QED systems are coherently connected by low-loss fiber channels. In such systems, quantum entanglement over the whole network can be created deterministically [21,22], instead of probabilistically [23]. Our achievement also paves the way for the study of many-body physics with atoms and photons in a coupled-cavities QED system, such as quantum phase transitions of light [24-31]."

-The authors could better emphasize what made this achievement possible. How the losses for instance are critical to observe this feature? Would have it been possible with current free-space cqed systems ?

We thank Reviewer #2 for this suggestion. The losses are indeed critical for the observation of dressed states of distant atoms with delocalized photons, since the coherent coupling rate between each cavity and the fiber has to be larger than the loss rates. All-fiber implementation allows us to reduce the loss in the channel

connecting the two cavities as low as 2%. If we were to couple two free-space cavities, it would have been extremely difficult to reduce the loss (which includes not only the pure losses but the inefficiency of the mode-matching) to this level. In order to emphasize these points, we have modified the following sentence in the introduction of the main text: "This is the first demonstration of coherent, reversible coupling between distant atoms, with a separation of the order of a meter, which is made possible by the all-fiber nature of the connection between the two cavities, with a loss as low as 2%."

- The authors use a relatively short fiber. I wonder what would be the effect of a much longer fiber, with a "significant" delay between the two cavities?

When the fiber length is long such that its free-spectral range is comparable or smaller than the coupling rate between the cavity and the fiber, the single-mode description of the fiber is no longer valid. To some extent, such an effect can be taken into account by extending the current single-mode model to the few-mode model. However, when the fiber is so long that its free-spectral range is significantly smaller than the fiber-cavity coupling rate, the fiber has to be treated as a continuum.

- At the end of the paper, saturation behaviors are given and fitted. The authors then give different quantities such as N_eff or n_sat without further details (sending then the reader to the SI). For the self-consistency of the main text, a few sentences to give the physical meaning will be welcome here.

N_{eff} has been defined and its physical meaning has been described at the end of the theory part in the main text. In order to give the physical meaning of n_{sat}, we have added the following sentence in the paragraph Reviewer #2 mentions: "Saturation behavior of a cavity QED system can be characterized with the so-called saturation photon number, which is a measure of the system nonlinearity."

- In the conclusion, the authors discuss the prospects. What should be the required improvements in their system (coupling, loss,...) to be able to tackle some proposals

(entangling or swapping gates) as proposed in the seminal reference [15] ?

As has been described in the original main text, it is required to reduce the number of atoms in each cavity to one for such future works. There are two major technical improvements required to observe the vacuum Rabi splitting between a single atom and the fiber-dark mode. Firstly, we need to further reduce the intrinsic losses (α_1, α_2, α_f) from the current values to achieve the strong coupling between a single atom and the fiber-dark mode. Secondly, in order to accumulate a sufficient amount of data within a realistic time, we need to actively stabilize the three cavities to the atomic frequencies and/or to extend the lifetime of the atom trap, since single atoms are loaded probabilistically. In contrast, ensembles of many atoms in the current study can be loaded deterministically. We have added the following sentences in the summary paragraph of the main text: "In order to extend our work to the construction of a fiber network of coherently coupled single-atom cavity QED systems, we are currently making technical improvements to the setup, *e.g.*, further reduction of the internal losses in the cavities, active stabilization of the three cavities, and extending the lifetime of the atomic trap [38]."

- Captions of the figures should be extended to be more self-consistent. For figure 1, the setup can be described. Captions of Figure 2 and 3 should include some comments to indicate to the readers what should be looked at the first sight. It will be a helpful guideline.

Following this suggestion, we have extended figure captions for Figs. 1-3.

- The Methods section is limited to the preparation of the atoms, without discussing the dipole trapping. This discussion is given in section B of the SI. I believe this section should better appear in the Methods section for the completeness of the main text.

Following this suggestion, we have moved the description of the dipole trapping from SI to the Methods section of the main text.

REVIEWERS' COMMENTS:

Reviewer #1 (Remarks to the Author):

The authors have satisfactorily addressed most of my comments and concerns. I also agree with their statement that fiber losses can be neglected when the coherent coupling rate between each cavity and the fiber is larger than the loss rates. However, in view of this insight, the corresponding sentence in the manuscript remains confusing: "Furthermore, the interaction of atoms with the fiber-dark mode can be utilized in quantum gates for distributed quantum computation that is insensitive to fiber losses [15] (as long as the normal mode description is valid, i.e., the coherent coupling rate between each cavity and the fiber is larger than the loss rates)." Instead, it would be more correct to state: "Furthermore, provided that the normal mode description is valid, i.e., if the coherent coupling rate between each cavity and the fiber is larger than the loss rates, the interaction of atoms with the resulting fiber-dark mode can be utilized in quantum gates for distributed quantum computation [15]." Finally, Ref. [36] should be cited prior to Refs. [34] and [35] because it was published earlier. Once these two points have been addressed, the paper is ready for publication.

Reviewer #2 (Remarks to the Author):

In the revised manuscript, the authors took into account all my initial comments and suggestions. Various points have been clarified accordingly. I definitely recommend publication in Nature Communications.

We thank both reviewers for their effort, for valuable comments, and for recommending publication of our manuscript.

The authors have satisfactorily addressed most of my comments and concerns. I also agree with their statement that fiber losses can be neglected when the coherent coupling rate between each cavity and the fiber is larger than the loss rates. However, in view of this insight, the corresponding sentence in the manuscript remains confusing: "Furthermore, the interaction of atoms with the fiber-dark mode can be utilized in quantum gates for distributed quantum computation that is insensitive to fiber losses [15] (as long as the normal mode description is valid, i.e., the coherent coupling rate between each cavity and the fiber is larger than the loss rates)." Instead, it would be more correct to state: "Furthermore, provided that the normal mode description is valid, i.e., if the coherent coupling rate between each cavity and the fiber is larger than the loss rates, the interaction of atoms with the resulting fiber-dark mode can be utilized in quantum gates for distributed quantum computation [15]." Finally, Ref. [36] should be cited prior to Refs. [34] and [35] because it was published earlier. Once these two points have been addressed, the paper is ready for publication.

Following the above comments by Reviewer #1, we have modified the sentence "Furthermore, the interaction of atoms with the fiber-dark mode can be utilized in quantum gates for distributed quantum computation that is insensitive to fiber losses [15] (as long as the normal mode description is valid, i.e., the coherent coupling rate between each cavity and the fiber is larger than the loss rates)" to "Furthermore, provided that the normal mode description is valid, i.e., if the coherent coupling rate between each cavity and the fiber is larger than the loss rates, the interaction of atoms with the resulting fiber-dark mode can be utilized in quantum gates for distributed quantum computation [15]." We have also reordered Refs. [34-36].
